# Supplementary material for: Towards Sustainable Shifts to Healthy Diets and Food Security in Sub-Saharan Africa with Climate-Resilient Crops in Bread-Type Products: A Food System Analysis
Source: Foods. 2022 Jan 6;11(2):135. doi: 10.3390/foods11020135 (PMC8774613; doi:10.3390/foods11020135)
Supplement: Supplementary file 1 [file foods-11-00135-s001.zip › foods-1501386-SI.pdf]

Supplementary materials

Table S1. Comparison of the cultivation requirements of the major cereal crops with those of the leading climate-resilient starch-rich crops which are cultivated in sub-Saharan Africa.

Common name	Proper name	Type of Crop	Cultivation habitat requirements	Tolerance to high temperatures	Tolerance to water-stress and drought	Other beneficial agronomic and crop attributes	References
Major cereals in SSA							
Bread wheat	*Triticum aestivum* L.	Cereal grain	Daily maximum temperature 22–30 °C and 600 mm rainfall. Ideally cool and moist with dry harvesting season. Loamy to sandy loam soils	Moderate	Poor drought tolerance		[1,2]
Dryland (upland) rice	*Oryza sativa* L.	Cereal grain	Minimum temperature > 15 °C. Rainfall 800–900 mm. Soil with a minimum of 15% clay	Moderate	Very poor		[3]
Maize	*Zea mays* L.	Cereal grain	Daily maximum temperate ≥ 23 °C. Rainfall 500–800 mm. 10–30% clay soils	Moderate	Poor drought tolerance	C4 plant*. The highest yield among cereals under optimal conditions	[2,4]
Climate-resilient crops							
Sorghum	*Sorghum bicolor* (L.) Moench	Cereal grain	Daily optimal maximum temperature 27–30 °C. Rainfall 450–650 mm. Prefers heavier soils than maize	More tolerant than maize	Drought-tolerant. The highest water use efficiency among cereals. Can withstand waterlogging	C4 plant*. Valuable fodder crop.	[2,5]
Pearl millet	*Pennisetum glaucum* (L.) R. Br.	Cereal grain	Daily optimal maximum temperature 23–30 °C. Rainfall 450–650 mm. Sandy or light loam soils	Highly tolerant	Less tolerant than sorghum	C4 plant*. Valuable fodder crop.	[2,6]
Grain amaranth	*Amaranthus* (several species)	Pseudocereal grain	Daily temperature maximum > 25 °C. Adapted to a wide variety of soils including marginal soils	Tolerant	Drought-tolerant. Cannot withstand waterlogging	C4 plant*. Leaves are used as a vegetable.	[7]
Cowpea	*Vigna unguiculata* (L.) Walp	Grain legume	Daily optimal maximum temperature 30 °C. Rainfall 400–700 mm. Prefers sandy soils but can be cultivated on a range of soils	Very tolerant	Drought-tolerant but prefers regular rainfall. High tolerance to waterlogging.	Fixes atmospheric nitrogen. Intercropping provides nitrogen to another crop. Used as green manure. Leaves are used as a vegetable.	[8]
Cassava	*Manihot esculenta* Crantz	Starchy root	Daily optimal maximum temperature 25–29 °C. Minimum rainfall 500 mm but can tolerate up to 5000 mm. Best cultivated in warm, humid tropical lowlands. Deep fertile light sandy loams or loamy sand soils preferred.	Tolerant	Highly drought-tolerant	The highest starch yield of all the crops under optimal conditions. Used as a perennial food security crop.	[9]

* C4 plants—their photosynthetic pathway cycles carbon dioxide to four-carbon sugars prior to entering C3 (Calvin cycle) making it more drought-tolerant.

Table S2. Annual production of climate-resilient crops and other crops and their commodity balances for Kenya, South Africa and Uganda [10].

			**Production (2016 data)**			**Commodity balances—crops primary equivalent (2013 data)**				
**Crop type**	**Crop**	**Country**	**Production (1000 tons)**	**Area planted (1000 ha)**	**Yield (tons/ha)**	**Crop (and products)**	**Production (1000 tons)**	**Imports (1000 tons)**	**Exports (1000 tons)**	**Domestic supply quantity (1000 tons)**
Starchy roots and tubers	Cassava	Kenya	571.8	46.5	12.3	Cassava and products	1112.4 (99.7) [1]	3.7 (0.3) [1]	< 0.1	1116.0
		South Africa	0	0	0		0	58.1 (100)	2.2	56.5
		Uganda	2885.4	945.6	3.1		2979.0 (101.5)	1.0 (< 0.1)	46.6	2934.2
	Sweet potato	Kenya	697.4	47.2	14.8	Sweet potatoes	1150.0 (100)	0	0	1150.0
		South Africa	61.5	21.5	2.9		65.0 (103.1)	0	2.0	63.0
		Uganda	2127.0	482.2	4.4		1810.0 (100)	0	0	1180.0
Cereals	Finger millet	Kenya	54.0	88.4	0.6	Finger millet and products	64.1 (88.9)	8.0 (11.1)	< 0.1	72.1
		South Africa	0	0	0		0	0	0	0
		Uganda	234.3	167.3	1.4		228.0 (100.1)	0.3 (0.1)	4.6	227.8
	Maize	Kenya	3330.0	2337.6	1.4	Maize and products	3339.1 (90.3)	112.2 (3.0)	5.7	3697.3
		South Africa	7778.5	1946.8	4.0		11,810.6 (112.7)	55.5 (0.5)	3036.4	10,479.6
		Uganda	2663.0	1149.0	2.3		2748.0 (96.2)	6.6 (0.2)	146.8	2857.9
	Sorghum	Kenya	117.0	184.7	0.6	Sorghum and products	138.5 (58.5)	104.3 (44.1)	6.1	236.7
		South Africa	70.5	48.5	1.5		147.2 (76.5)	55.8 (29.0)	60.5	192.4
		Uganda	414.6	398.1	0.8		299.0 (119.6)	4.3 (1.7)	53.2	250.1
	Wheat	Kenya	222.4	153.1	1.5	Wheat and products	485.8 (30.1)	1091.9 (67.7)	15.5	1612.3
		South Africa	1909.5	508.4	3.8		1870.0 (56.4)	1490.3 (45.0)	494.7	3316.1
		Uganda	23.0	14.5	1.5		20.0 (5.1)	464.1 (100)	88.1	396.0
Pulses (grain legumes)	Common beans (*Phaseolus*)	Kenya	728.2	1171.7	0.6	Common (*Phaseolus*) beans	529.3 (93.1)	47.8 (8.4)	43.3	568.7
		South Africa	34.4	34.4	1.0		60.2 (57.4)	51.3 (49.0)	6.5	104.9
		Uganda	1008.4	670.7	1.5		941.2 (103.1)	0.2 (< 0.1)	28.5	912.9
	Chickpeas	Kenya	2.0	5.6	0.4	Pulses (other) and products	228.5 (101.7)	4.1 (1.8)	22.9	224.7
		South Africa	0	0	0		23.5 ((77.3)	8.2 (27.0)	1.6	30.4
		Uganda	5.1	8.3	0.6		31.1 (100)	0.4 (1.2)	1.0	31.1
	Cowpeas	Kenya	146.8	227.8	0.6					

		South Africa	4.9	11.2	0.4					
		Uganda	12.9	26.3	0.5					
	Pigeon peas	Kenya	191.3	118.7	1.6					
		South Africa	0	0	0					
		Uganda	13.0	34.2	0.4					
Oilseed legumes	Groundnuts (peanuts)	Kenya	33.0	13.4	2.5	Groundnuts (shelled equivalents)	17.5 (66.5)	9.2 (35.0)	0.4	26.3
		South Africa	17.7	22.6	0.8		29.1 (53.7)	38.2 (70.5)	13.1	54.2
		Uganda	210.0	420.0	0.5		206.7 (98.5)	6.3 (3.0)	3.1	209.9
	Soya beans	Kenya	2.0	2.2	0.9	Soya beans	3.0 (32.3)	6.5 (70.0)	0.1	9.3
		South Africa	742.0	502.8	1.5		785.0 (107.3)	4.9 (0.7)	18.1	731.8
		Uganda	152.1	121.0	1.3		23.0 (109.0)	0.2 (0.9)	2.1	21.1

[1] Data in brackets are percentage of the domestic supply quantity.

Table S3. Approximate wholesale prices of climate-resilient crops and other crops and their flours versus wheat and wheat flour in Kenya, South Africa and Uganda in euros/ton in 2018.

Crop type	Crop	Kenya (KS 119 = 1 euro)	South Africa (ZAR 16.7 = 1 euro)	Uganda (UGX 4348 = 1 euro)
Starchy roots and tubers	Cassava (fresh roots)	223 [11] (131%)[a]	Not widely available	230 [12] (170%)[a]
	Cassava flour	294[f] (66%)[a]	Not widely Available	355[d] (54%)[a]
Cereals	Amaranth grain	1008[f] (270%)[a]	Not widely Available	575[d] (193%)[a]
	Amaranth flour	1680[f] (378%)[a]	Not widely Available	960[d] (145%)[e]
	Finger millet grain	675 [11] (180%)[a]	Not widely Available	364 [12] (122%)[a]
	Finger millet flour	756[11] (170%)[a]	Not widely available	661[d] (100%)[a]
	White maize grain	238 [11] (64%)[a]	210 [13], 145 [14] (55%)[a]	160[d] (54%)[a]
	White maize flour	378[f] (85%)[a]	268[c] (60%)[a]	299 [12] (45%)[a]
	Sorghum grain	410 [11] (110%)[a]	205 [13] (77%)[a] 211 [14]	218 [12] (73%)[a]
	Sorghum flour	420[f] (94%)[a]	565[c] (126%)[a]	366[d] (55%)[a]
	Wheat grain	374 [11] (100)	292 [13], 266 [14] (100)[a]	298 [15] (100)[a]
	Wheat flour	445[b] (100)	449[b] (100%)	661[d] (100)[a]
Pulses and oilseed legumes	Common (*Phaseolus*) beans	611 [11] (superior) (163%)[a]	Not widely available	494 [12] (superior) (166%)[a]
		536 [11] (basic) (143%)[a]	Not widely available	402 [12] (basic) (135%)[a]
	Cowpeas	652 [11] (174%)[a]	Not widely available	828 [12] (278%)[a]
	Groundnuts (peanuts)	1111 [11] (297%)[a]	1343 [13] (505%)[a]	839 [12] (282%)[a]
	Soya beans	504[f] (135%)[a]	403 [13], (152%)[a] 286 [14]	448 [12] (150%)[a]
	Toasted full fat soy flour	Not widely available	807[c] (180%)[a]	Not widely available

[a] Data in brackets are the percentage of wheat grain or flour cost on approx. 12% moisture basis, [b] Based on the cheapest retail price from the Internet, [c] Based on phone quotations, [d] Information from Prof. Yusuf Byaruhanga, Makerere University, Uganda, [e] Estimate based on the amaranth grain/amaranth flour differential in Kenya, [f] Information from Dr. Calvin Onyango, Kenya Industrial Research and Development Institute.

Table S4. Antinutrients, allergens and toxins in the climate-resilient food crops (cereal grains, pseudocereal grains, starchy roots and tubers and legume grains) produced in Kenya, South Africa and Uganda in comparison to bread wheat.

Component		Cereals				Pseudocereals	Roots and tubers		Pulses (grain legumes)			Oilseed legumes	
		**Wheat**	**Maize**	**Sorghum**	**Finger millet**	**Amaranth**	**Cassava**	**Sweet potato**	**Cowpeas**	***Phaseolus*-type beans**	**Bambara groundnuts**	**Soya beans**	**Peanuts (ground nuts)**
**Anti-nutrients**	Phytate	Present at levels of approx. 0.8–0.9% [16–18]				Present at high levels, approx. 3% [19]	Absent	Absent	Present at levels of approx. 0.6–1.5% [18,20]			Present at levels of approx. 0.6–1.5% [18,20]	
	Tannins	Absent	Absent	Present in some varieties only [16,21]		Absent	Absent	Absent	Present Varietal influence not clear [22]	Present Evident varietal influence [23]	Possibly present [24]	Absent	Absent
	Other polyphenols	Very low [25]	Normally low [25]	Many types and generally high [16,21]		Generally high [25]	Not known	Not known	Many types and generally high [26,27]			Rich in isoflavones [28]	Contains isoflavones [29]
**Allergens**		Common allergen. Associated with other sensitivities [30]	Uncommon	Uncommon	Not known	Not known	Not known but unlikely [31]	Not known	Protein allergens present [32]	Not known but likely	Not known but likely	Proteins can be allergenic Relatively rare but can be severe [33]	Proteins can be allergenic and allergenicity may be enhanced by processing. Common and severe [34]
**Toxins**		Gliadin-type epitopes trigger coeliac disease in susceptible individuals [35]	Absent	Very low levels in grain [21] Sprouted seeds may contain toxic levels of cyanogenic glycosides [36]	Sprouted seeds can contain high levels of cyanogenic glycosides [37]	Likely very low levels or absent	Often contains dangerously high levels of cyanogenic glucosides [38]	Likely very low levels or absent	Lectins present but normally destroyed by thermal food processing [26,32]			Lectins present in raw beans but largely inactivated by processing [39]	Contains lectins [40]

Table S5. Nutrient composition of the climate-resilient food crops produced in Kenya, South Africa and Uganda (cereal grains, pseudocereal grains, starchy roots and tubers and legume grains) in comparison to bread wheat (per 100 g as is basis) (except where stated otherwise, all the data are from [41]).

		Cereals				Pseudocereals	Starchy roots and tubers		Legumes				
									Pulses (grain legumes)		Oilseed legumes		
**Nutrient**	**Unit/ 100 g**	**Wheat (HRW)[a] (20072)**	**Maize (white) (20314)**	**Sorghum (20067)**	**Finger millet [41]**	**Amaranth (20001)**	**Cassava (11134)**	**Sweet potato[b] (11507)**	**Cowpeas (16062)**	***Phaseolus*-type beans (16037)**	**Bambara groundnuts [26]**	**Soya beans (16108)**	**Peanuts (groundnuts) (16087)**
Water	g	13.1	10.4	12.4	12.0	11.3	59.7	77.3	12.0	12.1	8.4	8.5	6.5
Energy	kJ	1368	1527	1377	1396	1554	667	359	1406	1411	1561	1866	2374
Carbohydrates, by difference (~starch)	g	71.2	74.6	72.1	75.0	65.3	38.1	20.1	60.0	60.8	58.9	30.2	16.1
Sugars	g	0.41	0.64	2.53	2.60	1.69	1.70	4.18	6.90	3.88	No data	7.3	4.7
Proteins	g	12.61	9.42	10.62	8.50	13.56	1.36	1.57	23.52	22.33	20.10	36.49	25.80
Lipids	g	1.54	4.74	3.46	1.40	7.02	0.28	0.05	1.26	1.50	5.90	19.94	49.24
Fiber, total dietary	g	12.2	7.3	6.7	15.2	6.7	1.8	3.0	10.6	15.5	3.7	9.3	8.5
**Essential amino acids**													
Lysine	g	0.335	0.265	0.229	0.257	0.747	0.044	0.066	1.591	1.280	1.327	2.706	0.926
Methionine	g	0.201	0.197	0.169	0.151	0.226	0.011	0.029	0.335	0.273	0.583	0.547	0.317
**Vitamins**													
Vitamin C	mg	0	0	0	0	4.2	20.6	2.4	1.5	No data	Trace	6.0	0
Thiamin	mg	0.383	0.385	0.332	0.480	0.116	0.087	0.078	0.853	0.775	0.300–0.470	0.874	0.640
Riboflavin	mg	0.112	0.201	0.096	0.170	0.200	0.048	0.061	0.226	0.164	0.100–0.140	0.870	0.135
Niacin	mg	5.464	3.627	3.688	1.280	0.923	0.854	0.557	2.075	2.188	1.800–2.000	1.623	1.207
Pantothenic acid	mg	0.954	0.424	0.367	No data	1.457	0.107	0.8	1.496	0.744	No data	0.793	1.767
Vitamin B6	mg	0.300	0.622	0.443	No data	0.591	0.088	0.209	0.357	0.428	No data	0.377	0.348
Folate, total	µg	38	19	20	No data	82	27	11	633	364	No data	375	240
Vitamin B12	µg	0	0	0	No data	0	0	0	0	0	0	0	0
Vitamin A, RAE	µg	0	0	0	No data	0	1	1100–1600 [42]	3	0	2	1	0
Vitamin E (alpha-tocopherol)	mg	1.01	0.49	0.50	2.20	1.19	0.19	0.26	0.39	0.02	No data	0.85	8.33
**Minerals**													
Calcium	mg	29	7	13	343	159	16	30	110	147	65	277	92
Iron	mg	3.19	2.71	3.36	8.70	7.61	0.27	0.61	8.27	5.49	3.30	15.70	4.58
Zinc	mg	No data	2.21	1.67	2.00	2.87	0.34	0.30	3.37	3.65	3.38	4.89	3.27

[a] Hard Red Winter

[b] proVit A data Orange-Fleshed

Table S6. Nutrient composition of the climate-resilient food crop flours (cereal grains, pseudocereal grains, starchy roots and tubers and legume grains) produced in Kenya, South Africa and Uganda in comparison to bread wheat flour (per 100 g, 10–12% moisture basis) (except where stated otherwise, all the data are from [11,41]).

		Cereal grains				Pseudocereals	Starchy roots and tubers		Legumes					
									Pulses (grain legumes)		Oilseed legumes			
**Nutrient**	**Unit/ 100 g**	**Refined wheat flour[a] (20129)**	**Degermed maize (White) (45255863-1)**	**Refined sorghum (20650)**	**Finger millet[c] [16]**	**Amaranth (45026829)**	**Cassava (11134)**	**Sweet potato[b] (11507)**	**Cowpeas[c] (16062)**	***Phaseolus*-type beans[c] (16037)**	**Bambara groundnuts[c] [26]**	**Full fat soya flour (16116)**	**Defatted soya flour (16117)**	**Defatted peanut flour (16099)**
Water	g	13.4	12.0	11.9	12.0	12.0	12.0	12.0	12.0	12.1	8.4	3.8	7.3	7.8
Energy	kJ	1510	1512	1492	1396	1541	1455	1390	1406	1411	1561	1836	1367	1368
Carbohydrates, by difference (~starch)	g	72.5	78.0	76.9	75.0	66.7	83.0	77.9	60.0	60.8	58.9	30.4	33.9	34.7
Sugars	g	0.31	0	No data	2.60	0	3.71	16.19	6.90	3.88	No data	7.61	16.42	8.22
Proteins	g	11.98	8.00	9.53	8.50	13.33	2.96	6.08	23.52	22.33	20.10	38.09	51.46	52.20
Lipids	g	1.66	2.00	1.24	1.40	6.67	0.61	0.19	1.26	1.50	5.90	21.86	1.22	0.55
Fiber, total dietary	g	2.40	2.00	1.90	15.2	10	3.92	11.61	10.6	15.5	3.7	9.7	17.5	15.8
**Essential amino acids**														
Lysine	g	0.231	No data	No data	0.257	No data	0.096	0.255	1.591	1.280	1.327	2.316	3.129	1.874
Methionine	g	0.210	No data	No data	0.151	No data	0.024	0.11	0.335	0.273	0.583	0.469	0.634	0.641
**Vitamins**														
Vitamin C	mg	0	0	0.60	0	4.00	44.96	9.30	1.5	No data	Trace	0	0	0
Thiamin	mg	0.080	No data	0.090	0.480	No data	0.190	0.302	0.853	0.775	0.300–0.470	0.412	0.698	0.700
Riboflavin	mg	0.060	No data	0.005	0.170	No data	0.105	0.236	0.226	0.164	0.100–0.140	0.941	0.253	0.480
Niacin	mg	1.000	No data	1.329	1.280	No data	1.864	2.157	2.075	2.188	1.800–2.000	3.286	2.612	2.7.00
Pantothenic acid	mg	0.440	No data	0.184	No data	No data	0.234	3.099	1.496	0.744	No data	1.209	1.995	2.744
Vitamin B6	mg	0.040	No data	0.068	No data	No data	0.192	0.810	0.357	0.428	No data	0.351	0.574	0.504
Folate, total	µg	33	No data	No data	No data	No data	59	43	633	364	No data	227	305	248
Vitamin B12	µg	0	No data	No data	No data	No data	0	0	0	0	0	0	0	0
Vitamin A, RAE	µg	0	0	0	No data	0	2.183	2420–3420 [42]	3	0	2	6	2	0
Vitamin E (alpha-tocopherol)	mg	0.40	No data	No data	2.20	No data	0.42	1.00	0.39	0.02	No data	1.98	0.12	0.05
**Minerals**														
Calcium	mg	15	0	6	343	133	35	116	110	147	65	188	241	140
Iron	mg	0.90	0	0.97	8.70	7.20	0.59	2.36	8.27	5.49	3.30	5.82	9.24	2.1
Zinc	mg	0.85	No data	0.47	2.00	No data	0.74	1.16	3.37	3.65	3.38	3.58	2.46	5.1

[a] Bread flour, unenriched, [b] proVit A data Orange-Fleshed, [c] Whole grain data

References

1. Department of Agriculture, Forestry and Fisheries, S.A. Wheat. Available online: https://www.dalrrd.gov.za/Portals/0/Brochures%20and%20Production%20guidelines/Wheat%20-%20Production%20Guideline.pdf (accessed on 1 February 2021).
2. Hadebe, S.T.; Modi, A.T.; Mabhaudhi, T. Drought Tolerance and Water Use of Cereal Crops: A Focus on Sorghum as a Food Security Crop in Sub-Saharan Africa. *J. Agron. Crop. Sci.* **2017**, *203*, 177–191. https://doi.org/10.1111/jac.12191.
3. Macdonald, C.I.; Zhanxi, L.; Hui, L.; Dongme, L. *Upland Rice Production in KwaZulu-Natal. Agri Update*; KZN Agriculture and Rural Development, Republic of South Africa, Pietermaritzburg, South Africa 2010.
4. Du Plessis, J. *Maize Production*; Directorate Agricultural Information Services: Pretoria, South Africa, 2003.
5. Department of Agriculture, Forestry and Fisheries, S.A. Sorghum Production Guide, 2010. Available online: www.arc.agric.za (accessed on 1 February 2021).
6. Department of Agriculture, Forestry and Fisheries, S.A. Pearl Millet. Available online: www.dalrrd.gov.za (accessed on 1 February 2021).
7. Department of Agriculture, Forestry and Fisheries, S.A. *Amaranthus Production Guideline*; Pretoria, South Africa, 2010.
8. Department of Agriculture, Forestry andFisheries, S.A. *Production Guidelines for Cowpeas*; Pretoria, 2014.
9. Department of Agriculture, Forestry and Fisheries, S.A. Cassava Production Guideline. Available online: www.nda.agric.za Pretoria, (accessed on 1 February 2021).
10. FAO FAOSTAT. Data. Available online: https://www.fao.org/faostat/en/#home (accessed on 1 March 2020).
11. Kenya Agriculture and Food Authority Monthly Wholesale Average Prices of Scheduled Food Crops. Available online: https://www.agricultureauthority.go.ke (accessed on 1 December 2018).
12. *Infotrade Uganda Food Prices*. Available online https://infotradeuganda.com/ (accessed on 1 December 2018).
13. SAGIS South African Grain Information Service, Import Parity Prices. Available online: http://www.sagis.org.za/parityprices.html (accessed on 1 November 2018).
14. SAFEX South African Futures Exchange. Available online: https://www.grainsa.co.za (accessed on 1 November 2018).
15. Uganda Radio Network Wheat prices in Uganda Soar. Available online: https://ugandaradionetwork.com (accessed on 1 November 2018).
16. Taylor, J.R.N. Millets: Their unique nutritional and health-promoting attributes. In *Gluten-Free Ancient Grains Cereals, Pseudocereals, and Legumes: Sustainable, Nutritious, and Health-Promoting Foods for the 21st Century*; Elsevier: Amsterdam, The Netherlands, 2017; pp. 55–103.
17. Febles, C.; Arias, A.; Hardisson, A.; Rodrı́guez-Alvarez, C.; Sierra, A. Phytic Acid Level in Wheat Flours. *J. Cereal Sci.* **2002**, *36*, 19–20. https://doi.org/10.1006/jcrs.2001.0441.
18. Lestienne, I.; Verniere, C.I.; Mouquet-Rivier, C.; Picq, C.; Trèche, S. Effects of soaking whole cereal and legume seeds on iron, zinc and phytate contents. *Food Chem.* **2005**, *89*, 421–425. https://doi.org/10.1016/j.foodchem.2004.03.040.
19. Sanz-Penella, J.; WRONKOWSKA, M.; Soral-Smietana, M.; Haros, M. Effect of whole amaranth flour on bread properties and nutritive value. *LWT* **2013**, *50*, 679–685. https://doi.org/10.1016/j.lwt.2012.07.031.
20. Ejigui, J.; Savoie, L.; Marin, J.; Desrosiers, T. Influence of Traditional Processing Methods on the Nutritional Composition and Antinutritional Factors of Red Peanuts (*Arachis hypogea*) and Small Red Kidney Beans (*Phaseolus vulgaris*). *J. Biol. Sci.* **2005**, *5*, 597–605. https://doi.org/10.3923/jbs.2005.597.605.
21. Serna-Saldivar, S.O.; Espinosa-Ramírez, J. Grain Structure and Grain Chemical Composition. In *Sorghum and Millets*; Elsevier BV: Amsterdam, The Netherlands, 2019; pp. 85–129.
22. Kayitesi, E. *Micronisation of Cowpeas: The Effects on Sensory Quality, Phenolic Compounds and Bioactive Properties*; University of Pretoria: Pretoria, South Africa, 2013.
23. Caldas, G.V.; Blair, M.W. Inheritance of seed condensed tannins and their relationship with seed-coat color and pattern genes in common bean (*Phaseolus vulgaris* L.). *Theor. Appl. Genet.* **2009**, *119*, 131–142. https://doi.org/10.1007/s00122-009-1023-4.
24. Nyau, V. Nutraceutical antioxidant potential and polyphenolic profiles of the Zambian market classes of bambara groundnuts (*Vigna subterranea* L. Verdc) and common beans (*Phaseolus vulgaris* L.); University of Cape Town: Cape Town, South Africa, 2013.
25. Dykes, L. Phenolic Compounds in Cereal Grains and Their Health Benefits. *Cereal Foods World* **2007**, *52*, 105–111. https://doi.org/10.1094/cfw-52-3-0105.
26. Duodu, K.G.; Apea-Bah, F.B. African Legumes: Nutritional and Health-Promoting Attributes. In *Gluten-Free Ancient Grains Cereals, Pseudocereals, and Legumes: Sustainable, Nutritious, and Health-Promoting Foods for the 21st Century*; Elsevier BV: Amsterdam, The Netherlands, 2017; pp. 223–269.
27. Lin, L.-Z.; Harnly, J.M.; Pastor-Corrales, M.S.; Luthria, D.L. The polyphenolic profiles of common bean (*Phaseolus vulgaris* L.). *Food Chem.* **2008**, *107*, 399–410. https://doi.org/10.1016/j.foodchem.2007.08.038.
28. Lee, C. Relative antioxidant activity of soybean isoflavones and their glycosides. *Food Chem.* **2005**, *90*, 735–741. https://doi.org/10.1016/j.foodchem.2004.04.034.
29. Chukwumah, Y.C.; Walker, L.T.; Verghese, M.; Bokanga, M.; Ogutu, S.; Alphonse, K. Comparison of Extraction Methods for the Quantification of Selected Phytochemicals in Peanuts (*Arachis hypogaea*). *J. Agric. Food Chem.* **2007**, *55*, 285–290. https://doi.org/10.1021/jf062148t.
30. Tatham, A.S.; Shewry, P.R. Allergy to wheat and related cereals. *Clin. Exp. Allergy* **2008**, *38*, 1712–1726. https://doi.org/10.1111/j.1365-2222.2008.03101.x.
31. Shewry, P.R. Tuber Storage Proteins. *Ann. Bot.* **2003**, *91*, 755–769. https://doi.org/10.1093/aob/mcg084.

32. Kumar, S.; Verma, A.K.; Das, M.; Jain, S.; Dwivedi, P.D. Clinical complications of kidney bean (*Phaseolus vulgaris* L.) consumption. *Nutrition* **2013**, *29*, 821–827. https://doi.org/10.1016/j.nut.2012.11.010.
33. Cordle, C.T. Soy Protein Allergy: Incidence and Relative Severity. *J. Nutr.* **2004**, *134*, 1213S–1219S. https://doi.org/10.1093/jn/134.5.1213s.
34. Boulay, A.; Houghton, J.; Gancheva, V.; Sterk, Y.; Strada, A.; Schlegel-Zawadzka, M.; Sora, B.; Sala, R.; Van Ree, R.; Rowe, G. A EuroPrevall review of factors affecting incidence of peanut allergy: Priorities for research and policy. *Allergy* **2008**, *63*, 797–809. https://doi.org/10.1111/j.1398-9995.2008.01776.x.
35. Di Sabatino, A.; Corazza, G.R. Coeliac disease. *Lancet* **2009**, *373*, 1480–1493. https://doi.org/10.1016/s0140-6736(09)60254-3.
36. Traoré, T.; Mouquet, C.; Icard-Vernière, C.; Traoré, A.; Trèche, S. Changes in nutrient composition, phytate and cyanide contents and α-amylase activity during cereal malting in small production units in Ouagadougou (Burkina Faso). *Food Chem.* **2004**, *88*, 105–114. https://doi.org/10.1016/j.foodchem.2004.01.032.
37. Chove, B.E.; Mamiro, P.R.S. Effect of germination and autoclaving of sprouted finger millet and kidney beans on cyanide content. *Tanzan. J. Health Res.* **2010**, *12*, 252–256. https://doi.org/10.4314/thrb.v12i4.51262.
38. Diop, A. *Storage and Processing of Roots and Tubers in the Tropics*; FAO: Rome, Italy, 1998.
39. Maenz, D.D.; Irish, G.G.; Classen, H.L. Carbohydrate-binding and agglutinating lectins in raw and processed soybean meals. *Anim. Feed. Sci. Technol.* **1999**, *76*, 335–343. https://doi.org/10.1016/s0377-8401(98)00215-6.
40. Rougé, P.; Culerrier, R.; Granier, C.; Rancé, F.; Barre, A. Characterization of IgE-binding epitopes of peanut (*Arachis hypogaea*) PNA lectin allergen cross-reacting with other structurally related legume lectins. *Mol. Immunol.* **2010**, *47*, 2359–2366. https://doi.org/10.1016/j.molimm.2010.05.006.
41. USDA National Nutrient Database for Standard Reference, Release 1 April 2018. Available online: https://ndb.nal.usda.gov (accessed on 10 December 2018).
42.van Jaarsveld, P.; Marais, D.W.; Harmse, E.; Nestel, P.; Rodriguez-Amaya, D. Retention of β-carotene in boiled, mashed orange-fleshed sweet potato. *J. Food Compos. Anal.* **2006**, *19*, 321–329. https://doi.org/10.1016/j.jfca.2004.10.007.
